# The Border-Land of Insanity

**Published:** 1885-06

**Authors:** W. B. Kesteven


### THE BORDER-LAND OF INSANITY.

BY

W. B. KESTEVEN, M.D. ST. AND., ENFIELD.

---

THE writer having for many years past been brought into contact with the undeveloped forms of insanity, has thought that hints as to their extent and means of arrest may not be uninteresting, albeit they lay no claim to novelty or originality.

Members of the medical profession are only too well aware of the existence of a numerous class of persons not recognised as lunatics, and not subject to legal restraint, but whose mental condition is such as to require medical supervision. These cases of incipient mental disease are sources of anxiety to their friends and their medical advisers, especially with reference to the enactments of the Lunacy Laws, as they are fraught with the danger of their infraction.

The importance can scarcely be exaggerated of early action in the treatment of these cases. The opportunity lost may lead to incurable mental disorder, involving present, and possibly future, generations in misery and crime ; for it is well known that in a large proportion of suicides and homicides an early vigilance might have averted the catastrophe.

At the very outset, however, of the derangements, the medical attendant, in his endeavour to cope with them, has to encounter an obstacle. There is an almost innate aversion in the minds of a large proportion of persons to admit the possibility of such an occurrence as insanity in their families. The bare suggestion will sometimes give offence, influenced by the superstitious feelings of past ages, that insanity conveys a stigma upon the character. Vagaries of conduct that should arouse the vigilance of friends are to be passed over as mere eccentricities. The more especially is this the case if there should happen really to exist an hereditary disposition in the direction of insanity. Any allusion to the fact is very likely to be pooh-poohed, with the cynical remark, "All are mad on some point!"

The conditions of life in modern civilization favour the development of all forms of Neuroses, and engender a psychological condition not far removed from mental disorder. That the nervous system is sooner exhausted than in past times is a fact with which medical practitioners are daily made familiar, and which has come to be recognised in the absolute necessity for a periodical holiday, to all engaged in the wear and tear of the battle of life, and which has taken concrete manifestation in the parliamentary enactment of "Bank Holidays," through the benevolent action of Sir John Lubbock.

One of the "outcomes" of this general morbid neurological condition is that of nervous dulness, which also is, doubtless, often due to hereditary causes.

"As we see external deformities produced and reproduced, generation after generation, by subjection of individuals to pernicious influences, so we see this nervous deformity produced and reproduced in certain families."

This condition of "nervous dulness" does not necessarily involve intellectual deficiency. The subject thereof may be "capable of reasoning, not directly injurious either to his neighbour or to his own well-being, yet entirely insusceptible to the finer subsidiary vibrations of the mental processes which underlie every mental operation. It is quite possible for such a man to do wrong without knowing it to be wrong. At the same time, it may be almost impossible to prove this fact, as evidence from his previous actions tending towards that proof may be entirely absent."*

Arising, also, out of this diffused neurotic atmosphere, is the undefinable BORDER-LAND of insanity, the limits of which shade off by degrees into pronounced insanity. The entire unconsciousness on the part of most of the subjects of impending mental disorder, is the source of great difficulty in preventive measures. There may, however, be the very reverse conditon, that of a dread of insanity, in itself imaginary, the climax of hypochondriasis, and the source of melancholia. This state is pointed out by Dr. Bucknill as existing in King Lear: "Conscious of his mental state, and even of its cause, he feels the goad of madness already urging him, and struggles and prays against it, and strives to push it aside. He knows its cause to be unbounded passion, and that to be kept in temper would avert it:

> 'Oh, let me not be mad, not mad, sweet heaven!
> Keep me in temper; I would not be mad!'" †

Dr. Bucknill contrasts the melancholy of Lear, with that of Jaques, who "cherished his melancholy, but, if he had thought fit to do so, retained the power to oppose, if not to repress it. Herein," adds Dr. Bucknill, "is the psychical difference between the sane and the insane melancholist;" with the essential difference, that "in the

* "Nervous Dulness," by W. H. Kesteven, *Journal Mental Science*, July, 1882.

† Bucknill's *Psychology of Shakspeare*, page 146.

one case there are evidences of cerebral disease, which are wanting in the other."

Among the phenomena indicative of the approach of mental disorder, the most important to be noticed is a change of character and disposition, and the supervention of eccentricities and habits in contrast to the usual moral bearing of the individual, the intellectual faculties being not at all, or but slightly, impaired. These indications should not be disregarded or lightly passed over. "Among the changes of character, also, indicative of the approach of mental disease, is the occurrence of aversion and antipathy towards former objects of respect or affection; a change so painful to those toward whom it is manifested, that it is usually the earliest to be noticed, and one that should by no means be disregarded. The change may be gradual—so gradual, that it may pass unnoticed for some time, or be difficult of detection even when suspected."

A precursor of insanity is to be feared in the occurrence of a dreamy absent manner in a person usually possessing a clear and practical energy. Such a person shunning society, becoming reserved and given to talking to himself, is not unlikely to be soon the subject of morbid illusions, or even of delusions. The indulgence of emotions disproportioned to their relations, and of an ill-regulated fancy; the habit of exaggeration and distortion of circumstances, will not be long in effecting a transformation into kleptomania, lying, &c. The subject of hypnotic, spiritualistic, and mesmeric trance, is for the time on the verge of insanity, and by repeated practices of the kind comes dangerously near to, if not actually within, the limits of lunacy, as by repeated relinquishment of his will he gradually loses the control thereof over actions which his judgment would condemn.

Of all the neuroses that tend towards mental unsoundness, a very common and very intractable one is hysteria in its countless manifestations. We may here find the mimicry of almost all diseases, bodily and mental. The dominant feature of hysteria is feebleness or abeyance of will, superadded, very often, to general weakness of mind. The patient is the easy dupe of any stronger mind, the action of which upon the more feeble one is, happily, often sufficient to effect marvellous restoration to health; while, on the other hand, morbid fancies and illusions are more often thereby aggravated. We know that in the hysterical "fit" the patient may be partly conscious of what is passing around her, although she is without the power to adapt herself to surrounding circumstances. In the asthenic mind of the subject of hysteria, moral control, being feeble to begin with, soon becomes lost; fancy runs riot; the judgment is deposed from its supremacy; a moral twist soon becomes apparent; deceit and imposition become habitual; the love of exaggeration dominates her words and actions, until, it may be unconsciously, the girl allows herself in lying and slandering.

"Picking and stealing" are often added to her list of delinquencies. The distinction between badness and incipient madness is very hard to be made out in such instances. It is not here implied that every hysterical girl is in imminent danger of becoming insane, but that unsubdued hysteria is prone to carry the patient close to the border-land of insanity.

A well-known cause that favours this condition, and of which the writer has witnessed several examples, is the excessive strain put upon the minds of young females in the brain-work of competitive examinations. Boys have, happily, a counterpoise to their brain-work in their athletic sports.

Of other causes that have come under the writer's observation—without the limits of actual insanity, but which have strongly tended to disturb mental balance—are, epilepsy; intemperance; ill-directed religious emotion; love of money and greed of gain; over-study; the puerperal state; cessation of menstruation. Among the most troublesome manifestations from these states, have been hypochondriasis, mental depression, melancholy, indolence, lying, kleptomania, erotomania, masturbation. Inordinate vanity, and uncontrollable temper, are states of mind which render the subjects thereof unfit for their domestic and social relations, while they may not actually be objects for legal control or restraint.

Where, as is often the case, there exists an hereditary disposition toward the neuroses, and toward insanity, the vagaries and eccentricities of such patients involve the oft-times perplexing question of certificates. The habitual drunkard, however, is the most hopelessly incurable and repulsive, though the epileptic is the most pitiable and most dangerous of all on the verge of mental disease; for of this come sudden, and seemingly unaccountably sudden, impulses to murder or suicide.

The practical point of the preceding observations is—What are the means to be adopted for the arrest of impending insanity?

They are simple, and may be summed up in a few words; but they must be carried out firmly, consistently, and persistently, to be efficient. They may be comprised under the following heads: *e.g.*, separation from all pernicious associations; bodily action and hygienic exercise; occupation of the mind by cultivation of the intellectual faculties; but, above all, by firm moral influence and control.

The first and most important step is, the breaking off of those associations which may have engendered or fostered the morbidly neurotic condition. Under the heading of bodily exercise may be enumerated walking, horse-riding, gymnastics, gardening, &c. In hysterical cases, cold affusion and cold baths are effective measures. By occupation of the intellectual faculties, the mind ceases to dwell upon trifling ailments and sensations. The patient is taught to cease to regard himself or herself as the centre of all care and anxiety, while, by judicious moral and religious influence, the prospect of a life of usefulness again opens out, while despondency disappears with turning mental and bodily health.

Great is the labour and patience that must be bestowed upon the efforts to remove the state of morbid feeling, to restore moral self-control, to disperse the clouds of darkness and despondency, and ensure a return to the sunshine of hope and life on the right side of the BORDER-LAND OF INSANITY.

